# Lower Expression of *CFTR* Is Associated with Higher Mortality in a Meta-Analysis of Individuals with Colorectal Cancer

**DOI:** 10.3390/cancers15030989

**Published:** 2023-02-03

**Authors:** Patricia Scott, Shuo Wang, Guillaume Onyeaghala, Nathan Pankratz, Timothy Starr, Anna E. Prizment

**Affiliations:** 1 Department of Biomedical Sciences, University of Minnesota Medical School, Duluth, MN 55812, USA; 2 Division of Epidemiology and Community Health, University of Minnesota School of Public Health, Minneapolis, MN 55455, USA; 3 Department of Laboratory Medicine and Pathology, University of Minnesota Medical School, Minneapolis, MN 55455, USA; 4 Department of Obstetrics, Gynecology and Women’s Health, University of Minnesota Medical School, Minneapolis, MN 55455, USA; 5 Division of Hematology, Oncology and Transplantation, University of Minnesota Medical School, Minneapolis, MN 55455, USA

**Keywords:** colorectal cancer, CFTR, survival, mortality, Cox proportional hazards regression, TCGA COADREAD

## Abstract

**Simple Summary:**

The ion channel gene *CFTR* is a tumor suppressor in colorectal cancer. It is well-established that individuals with cystic fibrosis, caused by biallelic germline mutations in *CFTR*, are at increased risk of developing colorectal cancer. A population of colorectal cancer patients with no known relationship to cystic fibrosis expresses reduced levels of *CFTR* in their tumors. This study aimed to determine if this population experienced increased mortality compared to those expressing higher levels of *CFTR*. Three independent datasets containing 1177 colorectal cancer patients were analyzed using Cox proportional hazards regression. Analysis of each study individually and meta-analysis of all three revealed an association between reduced *CFTR* expression and increased mortality. This association is potentially clinically significant because individuals with low *CFTR* expression may benefit from more aggressive treatment. Additionally, molecular therapies developed to treat cystic fibrosis by increasing *CFTR* activity may be applicable for colorectal cancer tumors expressing low levels of *CFTR*.

**Abstract:**

Individuals with cystic fibrosis (CF), caused by biallelic germline mutations in the cystic fibrosis transmembrane conductance regulator (*CFTR)*, have higher risk and earlier onset of colorectal cancer (CRC). A subset of CRC patients in the non-CF population expresses low levels of tumoral *CFTR* mRNA which may also cause decreased CFTR activity. To determine the consequences of reduced *CFTR* expression in this population, we investigated association of tumoral *CFTR* expression with overall and disease-specific mortality in CRC patients. *CFTR* mRNA expression, clinical factors and survival data from 1177 CRC patients reported in the Cancer Genome Atlas (TCGA) and Gene Expression Omnibus studies GSE39582 and GSE17538 were included. Log-transformed and z-normalized [mean = 0, standard deviation (SD) = 1] *CFTR* expression values were modeled as quartiles or dichotomized at the median. Univariate and multivariable Cox proportional hazards regression models were used to estimate hazard ratios (HR) and 95% confidence intervals (CI) for overall and disease-specific mortality in individual studies and meta-analyses. Analyses of each of the three individual datasets showed a robust association of decreased *CFTR* expression with increased mortality. In meta-analyses adjusted for stage at diagnosis, age and sex, *CFTR* expression was inversely associated with risk of overall death [pooled HR (95% CI): 0.70 (0.57–0.86)] and disease-specific death [pooled HR (95% CI): 0.68 (0.47–0.99)]. Associations did not differ by stage at diagnosis, age, or sex. Meta-analysis of overall death stratified by microsatellite instable (MSI) versus microsatellite stable (MSS) status indicated potential interaction between MSI/MSS status and *CFTR* expression, (*p*-interaction: 0.06). The findings from these three datasets support the hypothesis that low *CFTR* expression is associated with increased CRC mortality.

## 1. Introduction

The cystic fibrosis transmembrane conductance regulator (*CFTR*) gene encodes an ion channel expressed on the apical surface of luminal epithelia, including the lungs and intestine. The CFTR protein regulates transepithelial transport of Cl^−^ and HCO_3_^−^ ions to maintain water and salt homeostasis at the epithelial surface [1]. Biallelic germline loss of *CFTR* causes the hereditary life-shortening disease, cystic fibrosis (CF) [2]. The primary cause of mortality in CF is respiratory failure [3]; however, CF also causes clinically significant dysfunction in the intestine throughout the lives of CF patients including dysbiosis, inflammation, meconium ileus, and obstruction of the ileum and colon in adults and children [1,4]. As the lifespan of CF patients has improved, it has become apparent that another gastrointestinal manifestation of CF is increased risk of developing colorectal cancer (CRC) [5,6].

Initial evidence for the CF-CRC link came from a 20-year epidemiological study of more than 41,000 persons with CF in the United States (U.S.) [5]. The standardized incidence ratio for colon cancer was 6 times greater in non-transplanted CF patients compared to the U.S. general population, although the overall cancer risk was similar in these groups [5]. A recent meta-analysis of six additional population-based studies confirmed an increased risk of colon cancer among those with CF [6]. Clinical evidence for a direct connection between CF and CRC comes from endoscopic screening studies that found that adenomatous polyps appear earlier, and are more numerous and aggressive in individuals with CF than in the general population [7]. In line with this finding, the average age of CRC diagnosis among those with CF is 40 years, i.e., about 30 years lower than the average age in the general population [8].

This finding has been recapitulated in our mouse models of CF that directly tested the effects of loss of *CFTR* on intestinal tumorigenesis. In genetically modified mice with intestinal-specific deletion of *Cftr* [9], 61% of *Cftr*-deficient mice developed intestinal adenomas by the end of one year, as compared to none in the *Cftr* wild-type mice. When we added in an additional mutation in the *Apc* gene, a gene frequently mutated in human CRC, invasive carcinomas were observed, although they rarely develop in mice with only the *Apc* mutation [10].

These studies demonstrated that loss of *CFTR* via systemic germline mutations contributes to CRC in people with CF. However, loss of *CFTR* is also implicated in sporadic CRC without known connections to CF. Initially, we performed mutagenesis screens in genetically engineered mouse models that were designed to generate random somatic genetic alterations in the intestinal epithelium. Several of these screens identified *CFTR* as a candidate CRC driver gene, arguing that loss of CFTR activity may contribute to oncogenesis in sporadic human CRC [11,12,13].

In agreement with our animal studies, we found a significant association between *CFTR* mRNA expression in primary CRC tumors and 3-year disease-free survival of 90 persons diagnosed with stage II CRC [hazard ratio (HR) (95% confidence interval (CI)): 3.6 (1.20–10.77)], after adjusting for tumor location, differentiation, stage and microsatellite instability (MSI) status [10]. Our findings are consistent with findings from another study that reported lower *CFTR* mRNA and protein expression in CRC tumors versus normal tissue, and in metastatic CRC versus non-metastatic CRC [14]. 

These studies in humans and mice establish *CFTR* as a tumor suppressor gene in CRC. To better understand the contribution of reduced *CFTR* expression to the outcomes in individuals with CRC in the population without CF, we expanded on our earlier study and investigated the association between *CFTR* expression and survival of individuals with CRC in three independent cohorts: the COADREAD [COAD(COlon ADenocarcinoma) and READ (REctum ADenocarcinoma)] study from The Cancer Genome Atlas Program (TCGA) [15], and two studies with data deposited in the NCBI Gene Expression Omnibus (GEO) database, GSE39582 [16] and GSE17538 [17,18,19,20]. Together, these studies included 1177 persons diagnosed with CRC at stages II, III and IV, 374 of whom died. The data from these studies were analyzed individually and combined in meta-analyses to determine the risk of overall and disease-specific death associated with *CFTR* expression. These analyses were carried out to validate and extend the results of our pilot study, and to test our hypothesis that reduced *CFTR* expression is associated with worse outcomes in persons with CRC. 

## 2. Materials and Methods

### 2.1. Study Design

We examined overall mortality and disease-specific mortality of persons with CRC in relation to *CFTR* expression in the three studies: TCGA COADREAD, GSE39582 and GSE17538. The two GSE studies were selected because they have data on *CFTR* expression and overall survival (OS), and at least 100 participants.

### 2.2. TCGA COADREAD Study

mRNA expression data and related clinical information for CRC patients in the TCGA study were obtained from the cBioPortal for Cancer Genomics [21] data folder “Coadread_tcga_pan_can_atlas_2018”. mRNA expression data was extracted from the “data_RNA_Seq_v2_mRNA_median_all_sample_zscores” file [mean = 0 and standard deviation (SD) = 1]. mRNA sequence was generated using an Illumina HiSeq 2000 sequencer (Illumina Inc, San Diego, CA, USA) and processed using the RNAseqV2 pipeline, which uses MapSplice for alignment and RNA-Seq by Expectation-Maximization (RSEM) for quantification.

Clinical data for these patients were obtained from the “data_clinical_patient” file. This file contains information about cancer type (colon and rectal), age and stage at diagnosis, sex, and information about overall survival (OS) and disease-specific survival (DSS) and time of follow-up for each outcome. OS and DSS data were used to obtain overall and disease-specific deaths data used in our analyses in all three studies. After merging studies of individuals with CRC who had information about *CFTR* mRNA expression and OS (*N* = 577), we excluded individuals who were diagnosed at stage I (*N* = 103), and those with missing follow-up time or follow-up time equal to 0 (*N* = 21), resulting in 453 CRC cases (stages II–IV) available for the analysis of OS. For the analysis of disease-specific death 20 additional individuals were excluded because they did not have information about causes of death, leaving 433 CRC cases for this analysis. MSI and microsatellite stable (MSS) data were obtained from the “data_clinical_sample file”. In this study MSI was defined as an MSIsensor score ≥ 3.5, and MSS was defined as MSIsensor score < 3.5 [22].

Somatic mutation data was obtained from the UCSC Xena genomics platform (https://xena.ucsc.edu/, accessed on 21 January 2023) “dataset: somatic mutation (SNP and indel)—MC3 public version”. Cases reporting mutation status were merged with mRNA expression and clinical data from the cBioPortal to give a total of 300 cases.

### 2.3. GSE39582 and GSE17538 Studies

GSE39582 and GSE17538 were downloaded from the NCBI GEO database. In both studies, mRNA expression was measured by the Affymetrix Human Genome U133 Plus 2.0 Array (Thermo Fisher, Waltham, MA, USA) in a log2 scale and normalized using Robust Multi-array Average (RMA) [23]. We normalized mRNA expression to z-scores for all analyses.

#### 2.3.1. Patient and Clinical Information in the GSE17538 Study

This study includes 178 colon cancer cases from the Moffit Cancer Center, and 55 from the Vanderbilt University Medical Center (*N* = 233). Information about age, sex and stage at diagnosis, and data on OS and DSS, were obtained from the GSE17538-GPL570_series_matrix file. After excluding cases diagnosed at stage I (*N* = 29), 204 cases diagnosed at stages II-IV remained for the OS analysis, and 153 for the DSS analysis. MSI/MSS status was not reported in this study.

#### 2.3.2. Patient and Clinical Information in the GSE39582 Study

The study includes only persons with colon cancers (*N* = 566). Information about stage at diagnosis, age, sex, and MSI/MSS status, and data on OS (overall deaths, time of follow-up) were obtained from GSE39582_series_matrix. MSS was defined as “mmr-p” and MSI as “mmr-d”. No race information was available in GSE39582. Exclusion of colon cancer cases diagnosed at stage I (*N* = 37), and those with missing follow-up time or follow-up time equal to 0 (*N* = 9), resulted in 520 cases available for the OS analysis.

The data from all three studies, TCGA COADREAD, GSE17538 and GSE39582, are publicly available, so approvals by ethics committees were not required.

### 2.4. Statistical Analysis

Unless otherwise mentioned, all analyses were conducted using SAS (version 9.4, SAS Institute Inc, Cary, NC, USA). All statistical comparisons were performed at a two-sided significance level of 0.05 unless otherwise stated.

The demographic and clinical characteristics in each study were summarized. To estimate the risk of overall and disease-specific death across *CFTR* expression categories, we used Cox proportional hazards regression to estimate hazard ratios (HRs) and 95% confidence intervals (CIs). *CFTR* expression was analyzed as quartiles or dichotomized at the median to define high and low expressing groups. In the analysis of quartiles, *p*-trend was estimated by including the *CFTR* expression categories as an ordinal variable into the Cox proportional hazards model. Proportional hazards assumption was tested in each study using an interaction term between *CFTR* expression and follow-up time in relation to overall and disease-specific mortality and was not violated in any study. We conducted a univariate analysis and analysis a priori adjusted for age (continuous), sex (male versus female), and stage at diagnosis (II–IV). We did not adjust for race because the information was incomplete: GSE39582 did not collect information on race and 35% of TCGA COADREAD cases were missing data on race. Additionally, we conducted analysis further adjusted for MSI/MSS status in TCGA and GSE39582—the studies where this information was available, and for the colon/rectal subsites in TCGA.

To account for the possibility that death from causes other than CRC may be a competing event, we re-ran analyses of disease-specific deaths using the Fine-Gray sub-distribution hazard competing risk regression model [24].

OS and DSS across categories of *CFTR* expression were visualized using Kaplan-Meier plots and compared using a log-rank test (R statistical software, version 4.1.2, packages “survminer” and “survival”).

Risk of overall and disease-specific deaths associated with *CFTR* expression was also calculated using *CFTR* expression dichotomized at median (low versus high) in each study and in the meta-analyses (R statistical software, version 4.1.2, package “metafor”). The meta-analysis of overall deaths combined all three studies, whereas the meta-analysis of disease-specific deaths included only the two studies where this information was available, TCGA COADREAD and GSE17538. Meta-analyses were adjusted for stage at diagnosis, age and sex, and conducted using the fixed-effect model, since the HRs were in the same direction and *p*-values for heterogeneity between studies were not significant—0.54 for the analysis of overall death and 0.14 for the analysis of disease-specific death (Q-test).

Exploratory analyses were conducted stratified by age (dichotomized at median), sex (male/female), stage at diagnosis (II–IV), and MSI/MSS (MSI versus MSS) in the meta-analyzed data. To perform this analysis, we first stratified each dataset by the variable of interest and then meta-analyzed the association between *CFTR* expression and death (overall or disease-specific) in each stratum. MSI/MSS status was only available in the TCGA COADREAD and GSE39582, so only these two studies contributed data to this analysis.

## 3. Results

### 3.1. Descriptive Analysis

We analyzed data from three independent studies that contained *CFTR* expression data and associated survival data: TCGA COADREAD, GSE17538, and GSE39582. Median age and male/female ratios were similar in these three studies (Table 1), as were the ranges between z-scores for highest and lowest *CFTR* gene expression: 5.95 for TCGA, 4.61 for GSE17538, and 5.93 for GSE39582 (Supplementary Table S1). There was some difference in the proportions of people diagnosed with Stage IV versus Stage II, with the GSE17538 study having a higher proportion of Stage IV and lower proportion of Stage II compared to the TCGA COADREAD and GSE39582 studies (Table 1).

### 3.2. CFTR Expression and the Risk of Overall Death

We used three Cox proportional hazards regression models to analyze the association of *CFTR* expression with overall death. Model 1 was an unadjusted model, Model 2 was adjusted for stage at diagnosis, age and sex, and Model 3 was additionally adjusted for MSI/MSS status (Table 2).

In Model 1 in the TCGA study, higher *CFTR* expression modeled as quartiles was significantly associated with lower risk of death across quartiles (*p*-trend = 0.04) (Table 2). There was also an indication of higher *CFTR* expression associated with lower risk in Model 2, but this association did not reach statistical significance. The TCGA study included both colon and rectal cancer cases. No significant associations were observed when these analyses were limited to only those with colon cancer (Supplementary Table S2). The sample size was too limited to examine rectal cancer separately, as there were only 12 deaths among 87 rectal cancer cases.

In the GSE17538 and GSE39582 studies, there was a significantly lower risk of overall death for those with higher *CFTR* expression in all models (Table 2). For instance, for the highest versus lowest quartile, in Model 2, HRs (95% CI) were 0.31 (0.16–0.57, *p*-trend < 0.01) in GSE17538, and 0.64 (0.42–0.97, *p*-trend = 0.03) in GSE39582.

We also conducted Model 2 analysis after dichotomizing patients into high and low categories using median *CFTR* expression. There was an indication of association between higher *CFTR* expression and lower risk of death for all three studies. However, the association was significant only in GSE17538, HR (95% CI): 0.57 (0.37, 0.87) (Supplementary Table S3).

**Figure 1:**
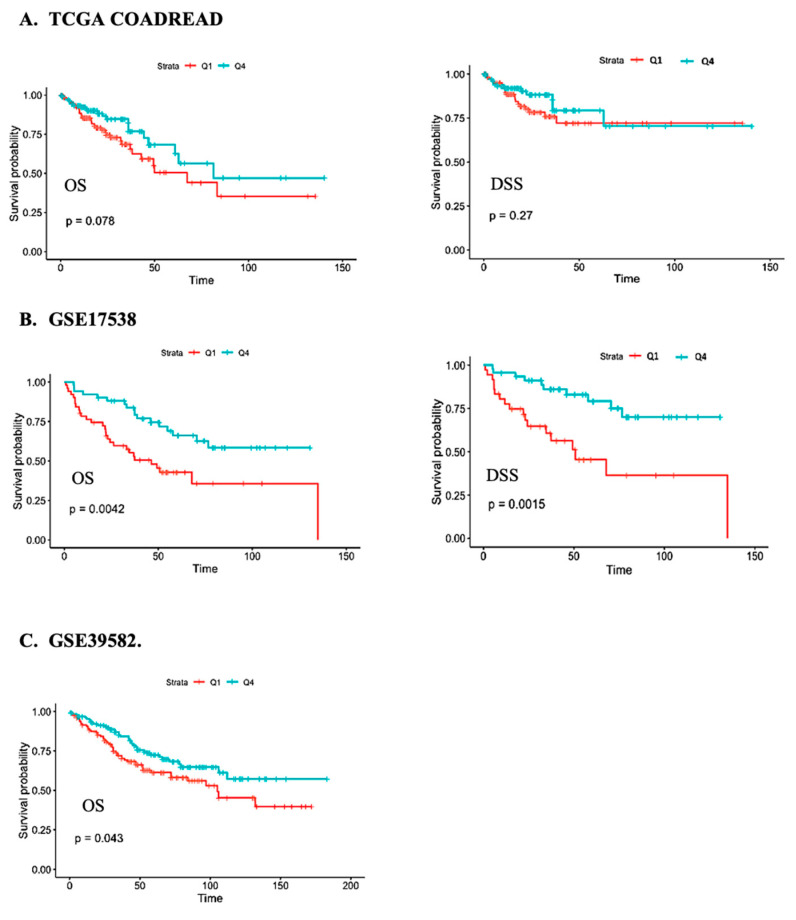
Kaplan-Meier survival analysis of the overall survival (OS) and disease-specific survival (DSS) of individuals with CRC in the (**A**) TCGA COADREAD, (**B**) GSE17538, and (**C**) GSE39582 studies in relation to *CFTR* expression for the fourth (green) versus first (red) quartile.

In agreement with the findings from Cox proportional hazards regression models, Kaplan-Meier analysis indicated that OS was better in patients expressing the highest levels of *CFTR* (quartile 4) compared to patients expressing the lowest levels of *CFTR* (quartile 1). Log-rank *p*-values were 0.078, 0.042 and 0.043 for TCGA, GSE17538, and GSE39582, respectively (Figure 1).

**Figure 2:**
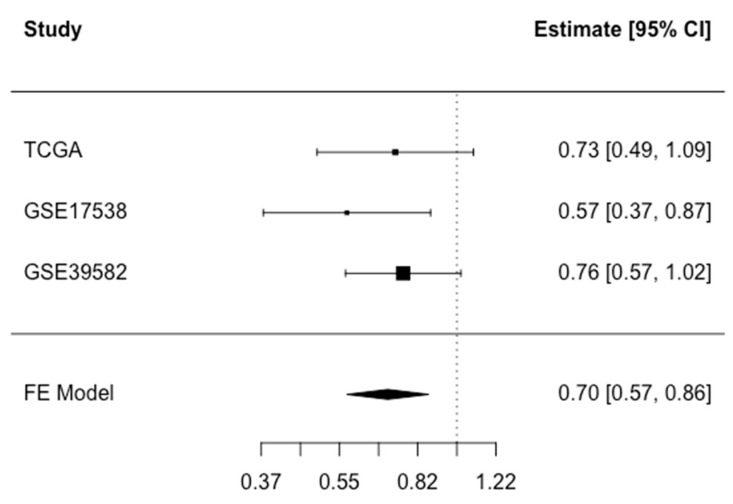
Meta-analysis of overall death associated with *CFTR* expression dichotomized at median in the TCGA COADREAD, GSE17538, and GSE39582 studies. Fixed Effect (FE) meta-analysis HR (CI 95%): 0.70 (0.57–0.86) for high versus low *CFTR* expression. Test for heterogeneity *p* = 0.54.

Finally, we performed a meta-analysis of the findings from all three studies to calculate the association of *CFTR* expression with overall death. In this meta-analysis, high *CFTR* expression (dichotomized at median) was associated with decreased overall death with HR (95% CI): 0.70 (0.57–0.86) (Figure 2). The associations did not differ significantly across age, sex or stage (Supplementary Table S4). There was a suggestive interaction between *CFTR* expression and MSI/MSS status, with an inverse association observed for MSS tumors only (*p*-interaction = 0.06), but the sample size for those with MSI status was too limited for analysis of this group (Supplementary Table S4).

### 3.3. CFTR Expression and the Risk of Disease-Specific Death

**Figure 3:**
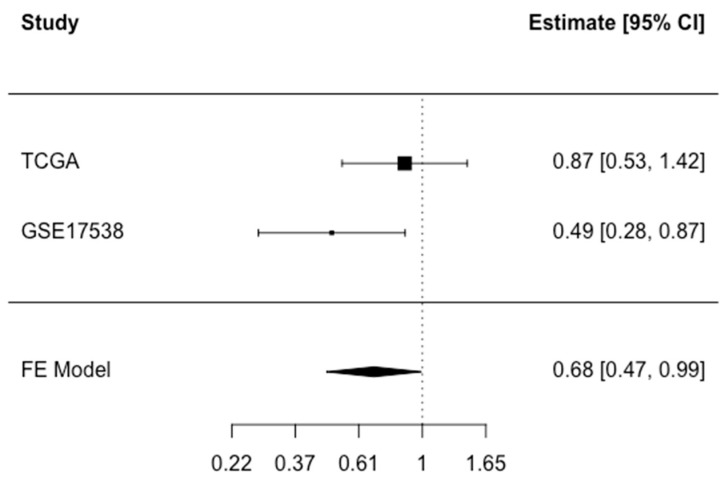
Meta-analysis of disease-specific death associated with *CFTR* expression dichotomized at median in TCGA COADREAD and GSE17538. Fixed effect (FE) meta-analysis HR (95% CI): 0.68 (0.47–0.99) for high versus low CFTR expression. Test for heterogeneity *p* = 0.14.

Information about disease-specific survival (DSS) was available in TCGA COADREAD and GSE17538. There was an indication of inverse associations between *CFTR* expression and disease-specific death in all models in both studies (Table 3, Supplementary Table S5). However, the associations were only statistically significant in GSE17538. For example, in Model 2, for the highest versus lowest quartile, HR (95% CI): 0.22 (0.09-, 0.47, *p*-trend < 0.01). Analyses that treated non-CRC deaths as competing events showed similar findings to the cause-specific hazard analysis (Supplementary Table S6). In the meta-analysis that pooled the findings from both studies, HR (95% CI) was 0.68 (0.47, 0.99) for high versus low *CFTR* expression (Figure 3). In meta-analyses stratified by stage at diagnosis, sex, or age, the associations did not differ significantly across subgroups (Supplementary Table S6). Information about disease-specific death and MSI/MSS status was only available for the TCGA dataset, thus the limited sample size (6 disease-specific deaths among those with MSI) precluded stratification by MSI/MSS status.

### 3.4. Somatic Mutations in CFTR in CRC

Somatic mutations in *CFTR* may contribute to decreased *CFTR* mRNA levels and/or to increased mortality. Mutational status based on whole exome sequencing (which captures a subset of *CFTR* mutations) was reported in the TCGA COADREAD study but not GSE17538 and GSE39582. Of the cases analyzed in our study, 300 reported data on somatic mutations. In these 300 cases, 22 non-synonymous *CFTR* mutations were found in 17 cases, i.e., 5.7% of cases. Eight mutations were categorized as loss of function (LOF) (loss or gain of stop codon, splice and frameshift mutations), while 14 were missense mutations. Two of the LOF mutations, R851*and E1104*, are also found in the CFTR2 database where they are characterized as CF-causing mutations (Table 4). We did not detect a significant correlation between *CFTR* mutation status (i.e., the presence of at least one *CFTR* mutation) and *CFTR* expression, although this analysis needs to be repeated in a larger study in the future. For all cases carrying any of the 22 mutations, the Point-Biserial correlation coefficient (PBCC) between *CFTR* mutation status and *CFTR* expression was r = −0.08 and for cases carrying LOF mutations, PBCC r = −0.0470. The number of cases with mutations and number of deaths among these cases were insufficient to carry out survival analysis. An additional complication was that most of the cases containing *CFTR* mutations (13 of 17 cases) belong to either the miscrosatellite instable (MSI) or polymerase epsilon (POLE) CRC subtypes. Both subtypes are characterized by deficiencies in DNA repair and tumors typically have mutation burdens of 1000 or more mutations per tumor, so it is difficult to estimate the functional impact of any one mutation.

## 4. Discussion

Epidemiological studies have identified a strong association between CF patients and CRC risk, with CF patients having a much higher CRC rate and earlier age of onset. There are also more than 10 million CF carriers (a single mutated allele) in the U.S. population. CF carriers have decreased CFTR protein function compared to the general population [25] and may also be at increased risk. In addition, there is a subset of individuals with CRC who express reduced levels of *CFTR* in their tumors. It is unclear, however, if expression of *CFTR* is associated with overall and disease-specific survival in sporadic CRC with no known relationship to CF. To address this question, we carried out an analysis of the association of *CFTR* expression with overall and disease-specific risk of death in 1,177 persons with CRC from three studies—TCGA COADREAD, GSE17538, and GSE39582. Analysis of each individual study showed an inverse relationship between *CFTR* expression and mortality, with the strongest association in GSE17538. In meta-analyses of these studies, we found that high versus low *CFTR* mRNA expression was significantly associated with a 30% decreased risk of overall death and 32% decreased risk of disease-specific death after adjusting for age, sex and stage at diagnosis when dichotomizing CRC patients by median *CFTR* expression. The associations did not differ by age, sex, or stage. *CFTR* expression was also similar across stages suggesting that the changes in *CFTR* expression occur at earlier stages of CRC.

This analysis is consistent with the findings of our initial study of 90 patients with stage II CRC [10]. In that study, we reported that lower *CFTR* expression in CRC tumors was associated with a 29% decrease in 3-year disease-free survival for the 27% of patients with lower *CFTR* expression versus the 73% with higher expression [10]. The patient population in that initial study is comparable to the populations in TCGA COADREAD, GSE17538 and GSE39582 included in the current study in age and sex distribution [age: 73.4 (34.6–95.1 years); male: 42 (46.7%)] [26]. However, the initial study included only 90 persons with CRC and examined only cases diagnosed at stage II, while the current study examined 1177 cases diagnosed at stages II-IV. Thus, our current study with a much larger sample size extends our initial work by showing that similar associations exist for stages II-IV for overall and disease-specific death.

Further, we found a potential interaction between MSI/MSS status and *CFTR* expression (*p* = 0.06). The association between reduced *CFTR* expression and increased overall death appears to exist among those with MSS but not with MSI status. This result suggests that a differential effect of *CFTR* high versus low expression in MSS cases most likely drives the inverse association of *CFTR* expression with increased mortality. The finding of the association for the MSS cases is consistent with the findings from several studies which report that diminished CFTR activity leads to the activation of Wnt/β-catenin signaling—a fundamental pathway in CRC development [10,27,28]. Because the sample size for MSI cases is limited (31 deaths among 126 cases), we cannot conclude whether there is association among this group. Nor could we examine the interaction with MSI/MSS status in relation to disease-specific death, since the CRC cases with MSI/MSS status and disease-specific death were available in the TCGA study only (number of CRC deaths = 6).

The biological basis for association between low *CFTR* expression and poor survival of individuals with CRC is unknown. However, it is known that loss of *CFTR* due to germline mutations in individuals with CF causes severe manifestations in the gastrointestinal tract that are potentially oncogenic, including intestinal obstruction, dysbiosis, and inflammation [1,4]. Experimentally, loss of *CFTR* in animal and cell culture models is associated with activation of the oncogenic Wnt/β-catenin [27,28] and NF-κB pathways [29,30].

A number of factors may contribute to differential expression of *CFTR* in CRC tumors. In CF carriers with CRC, *CFTR* expression may be limited due to the presence of a single mutant allele. However, the estimated frequency of CF carriers among individuals with CRC is ~6% [31], which is likely not sufficient to explain the association between *CFTR* expression and mortality.

*CFTR* mRNA levels could also be affected by somatic genetic alterations. The TCGA COADREAD study reports somatic mutations identified from whole exome sequencing. In the cases analyzed in our study, 22 mutations were reported in 17 of 300 cases, or 5.6% including 8 classified as LOF and 14 classified as missense. We did not detect correlation between *CFTR* mutation status and *CFTR* expression although this analysis needs to be repeated in a larger study. Because of the relatively small number of mutations and lack of correlation with expression it is unlikely these mutations are driving the inverse association that we report between *CFTR* expression and mortality.

*CFTR* levels may also be affected by many other processes including alterations in signaling pathways that control transcription factors regulating *CFTR* expression, large chromosomal changes, and epigenetic changes including DNA methylation or histone acetylation. *CFTR* transcription in the normal intestine is regulated by a complex array of factors acting at upstream and intronic enhancers as well as at promoters. In particular, cis regulatory elements within intron 1 and intron 11 are involved in intestinal-specific regulation. A number of factors promote intestinal-specific transcription through interaction with these elements [32]. Among these, CDX2 is of particular interest because loss of CDX2 is associated with CRC [33,34]. In addition, hypermethylation of the *CFTR* promoter and consequent downregulation have been associated with several cancers, including lung, breast and CRC [35,36,37]. However, the relationship between these factors, *CFTR* low expression and CRC survival remains to be determined.

Our study is the first to examine the association between the *CFTR* expression and CRC survival in a large study of 374 overall deaths among 1177 CRC cases. A strength of our study is that we were able to account for stage, while a limitation is that we could not account for treatment or for lifestyle factors such as diet, smoking and obesity. We also could not analyze the effect of race because data was only available in two out of three studies and was severely limited in the TCGA COADREAD study where 35% of cases were missing information on race. Given the higher CRC incidence and CRC deaths among the Black population, in the future, it will be important to determine if decreased *CFTR* expression is associated with poor survival in this population as well. Finally, mRNA expression in the TCGA and GEO studies was determined using different platforms: RNA sequencing in TCGA COADREAD and RNA microarrays in the GSE studies. However, we normalized mRNA expression as z-scores, and the range of *CFTR* z-score measures was similar in all three studies. The ranges between the highest and lowest *CFTR* z-scored expression on the log scale were comparable across the studies. We also modeled z-scored expression as quartiles or dichotomized at the median because this presentation is less sensitive to different methods of measurements and to outliers compared to continuous variable presentation. Importantly, the associations were in the same direction in all three studies and there was no evidence of heterogeneity across studies, which provides further credibility to our findings.

We examined the association of loss of mRNA expression with survival, and association of somatic *CFTR* mutations with *CFTR* expression. However, it was beyond the scope of this study to investigate the effects of germline mutations in CF carriers. In future work it will be important to identify CRC cases with CFTR germline mutations to provide a more complete picture of the impact of loss of CFTR activity on survival. In addition, new CF modulator therapies which are mutation-specific may be applicable to these cases. Finally, identification of the spectrum of mutations found in CRC may aid in understanding which CFTR functions are important for tumor suppression.

## 5. Conclusions

In summary, consistent with our hypothesis, we have found an association between higher *CFTR* expression and lower risk of overall and disease-specific death in individuals with CRC. This association is potentially clinically significant because individuals with low *CFTR* expression may benefit from more aggressive treatment. In addition, the last 10 years have seen the development of highly effective modulator therapies for the treatment of CF. These small molecule therapies restore function of mutant CFTR proteins and thus treat the underlying cause of CF rather than the symptoms and effects [38]. Some of these therapies may be applicable for treatment of *CRC* tumors expressing low levels of *CFTR*. CF modulators may be potentially useful in several CRC situations:

1. In CRC which harbor known CF-causing mutations, modulators may be available to restore function of specific mutations. For example, therapies currently under development to promote readthrough of premature stop codons could be used to rescue CFTR function in cases harboring nonsense mutations [39].

2. Rare CF-causing mutations are tested for response to modulators using patient-derived cell lines and organoids. Similar reagents could be used to test uncharacterized *CFTR* mutations in CRC for response to modulators [40].

3. Modulators such as ivacaftor that increase CFTR ion channel activity by increasing time in the open conformation could potentially compensate for low expression of *CFTR* mRNA by increasing the activity of the relatively small amount of protein that is made [41].

Our study highlights the importance of understanding mechanisms of downregulation of *CFTR* in CRC. Our future studies will broaden our work to address this question.

## Figures and Tables

**Table 1:** Characteristics of individuals with colorectal cancer (CRC) in the TCGA COADREAD, GSE17538, and GSE39582 studies.

Characteristics	TCGA COADREAD	GSE17538	GSE39582
Total number (*N*) of CRC cases ^1,2^	453 (colon: 269; rectal: 87; missing: 97)^2^	204	520
Age, median (range), years	67 (31–90)	65 (23–94)	68 (22–97)
Female, N (%)	213 (47.02)	101 (49.51)	231 (44.42)
Race, N (%) White Black Asian Hispanic Other/Missing	228 (50.33) 54 (1.92) 12 (2.65) — 159 (35.01)	177 (86.76) 13 (6.37) — 2 (0.98) 12 (5.88)	—
Stage at diagnosis, N (%) 2 3 4	207 (45.70) 165 (36.42) 81 (17.88)	72 (35.29) 76 (37.25) 56 (27.45)	258 (49.62) 203 (39.04) 59 (11.35)
MSI/MSS status,^3^ N (%) MSI MSS Unknown/Missing	64 (14.13) 381 (84.11) 8 (1.77)	—	62 (11.92) 412 (79.23) 46 (8.85)
Follow-up time median (range), months	22.36 (0.20–148.01)	41.49 (0.43–134.86)	52 (1–201)
No. of deaths from all causes/CRC	104/65	89/54	181/UK

Abbreviations: TCGA COADREAD, the Cancer Genome Atlas (colon and rectal adenocarcinoma); GSE dataset, GEO (Gene Expression Omnibus) Series dataset; MSI, Microsatellite Instable; MSS, Microsatellite stable; N, number; UK, unknown. ^1^ CRC cases with stage 1 at diagnosis were excluded. ^2^ TCGA COADREAD dataset included both colon and rectal cancer cases. GSE17538 and GSE39582 included colon cancer cases only. ^3^ Information about MSS/MSI status was available in TCGA COADREAD and GSE39582, but not in GSE17538.

**Table 2:** Hazard ratio (HR) and 95% confidence interval (CI) for overall death in relation to *CFTR* mRNA expression, presented as quartiles in individuals with CRC/colon cancer in the TCGA COADREAD, GSE17538, and GSE39582 studies.

Dataset	HR (95% CI) across Quartiles (Q1 is Reference)				*p*-Trend ^4^
**TCGA COADREAD**					
*CFTR* Expression	Q1: −1.54–(−0.74)	Q2: −0.73–(−0.07)	Q3: −0.06–0.49	Q4: 0.49–4.41	
Overall death (*N*)	33	27	23	21	
Person-years (months)	3005	3404	3559	3111	
Model 1 ^1^ (*N* = 453) Model 2 ^2^ (*N* = 453) Model 3 ^3^ (*N* = 356)	1.00 (Reference) 1.00 (Reference) 1.00 (Reference)	0.74 (0.45, 1.24) 0.76 (0.45, 1.27) 0.74 (0.40, 1.37)	0.59 (0.35, 1.00) 0.55 (0.32, 0.95) 0.47 (0.24, 0.93)	0.61 (0.35, 1.05) 0.77 (0.44, 1.34) 0.71 (0.36, 1.40)	0.04 0.16 0.17
**GSE17538**					
*CFTR* expression	Q1: −1.53–(−0.74)	Q2: −0.64–(−0.07)	Q3: −0.04–0.40	Q4: 0.50–3.49	
Overall death (*N*)	29	22	21	17	
Person-years	1895	2182	2211.27	2926	
Model 1 ^1^ (*N* = 204) Model 2 ^2^ (*N* = 204)	1.00 (Reference) 1.00 (Reference)	0.69 (0.39, 1.20) 0.49 (0.27, 0.87)	0.66 (0.38, 1.17) 0.48 (0.26, 0.86)	0.43 (0.24, 0.79) 0.31 (0.16, 0.57)	<0.01 <0.01
**GSE39582**					
*CFTR* expression	Q1: −4.31–(−0.39)	Q2: −0.39–0.28	Q3: 0.29–0.71	Q4: 0.71–1.62	
Overall death (*N*)	51	46	46	38	
Person-years	6992	7187	7801	7992	
Model 1 ^1^ (*N* = 520) Model 2 ^2^ (*N* = 520) Model 3 ^3^ (*N* = 474)	1.00 (Reference) 1.00 (Reference) 1.00 (Reference)	0.86 (0.58, 1.28) 0.87 (0.58, 1.30) 0.92 (0.60, 1.42)	0.82 (0.55, 1.22) 0.77 (0.52, 1.16) 0.76 (0.49, 1.19)	0.65 (0.43, 0.99) 0.64 (0.42, 0.97) 0.60 (0.38, 0.96)	0.05 0.03 0.02

Abbreviations: TCGA COADREAD, the Cancer Genome Atlas (colon and rectal adenocarcinoma); GSE dataset, GEO Series dataset; MSI, Microsatellite Instable; MSS, Microsatellite stable; *N*, number; Q1, Q2, Q3 and Q4, quartiles. Note: Only stages 2–4 were included in all the studies. TCGA COADREAD included both colon and rectal cancer cases. GSE17538 and GSE39582 included colon cancer cases only. ^1^ Model 1: unadjusted. ^2^ Model 2: adjusted for stage at diagnosis, age and sex. ^3^ Model 3: adjusted for stage at diagnosis, age, sex, and MSI/MSS status. For the TCGA analysis of individuals with CRC, this model was also adjusted for CRC subsite (colon/rectal). MSI/MSS status was not available for GSE17538. The total number of cancer cases is lower in this model than in Models 1 and 2 in the corresponding analyses because of missing data on MSI/MSS status and CRC subsite. ^4^ *p*-trend was computed by including CFTR quartiles as an ordinal variable into the corresponding model.

**Table 3:** Hazard ratio (HR) and 95% confidence interval (CI) for disease-specific death in relation to *CFTR* mRNA expression, presented as quartiles among individuals with CRC/colon cancer in the TCGA COADREAD and GSE17538 studies.

Dataset	HR (95% CI) across Quartiles (Q1 is Reference)				*p*-Trend ^4^
**TCGA COADREAD**					
*CFTR* Expression	Q1: −1.54–(−0.74)	Q2: −0.73–(−0.07)	Q3: −0.06–0.49	Q4: 0.49–4.41	
Disease-specific death (*N*)	20	13	18	14	
Person-years (months)	2855	3241	3368	2897	
Model 1 ^1^ (*N* = 433) Model 2 ^2^ (*N* = 433) Model 3 ^3^ (*N* = 336)	1.00 (Reference) 1.00 (Reference) 1.00 (Reference)	0.60 (0.30, 1.21) 0.54 (0.27, 1.10) 0.45 (0.19, 1.08)	0.77 (0.41, 1.46) 0.59 (0.31, 1.13) 0.45 (0.20, 1.04)	0.69 (0.35, 1.37) 0.74 (0.37, 1.48) 0.62 (0.26, 1.49)	0.40 0.38 0.39
**GSE17538**					
*CFTR* expression	Q1: −3.13–(−0.54)	Q2: −0.46–0.29	Q3: 0.29–0.68	Q4: 0.69–1.48	
Disease-specific death (*N*)	18	13	13	10	
Person-years	1305	1470	1498	2695	
Model 1 ^1^ (*N* = 153) Model 2 ^2^ (*N* = 153)	1.00 (Reference) 1.00 (Reference)	0.69 (0.33, 1.42) 0.68 (0.33, 1.42)	0.68 (0.33, 1.40) 0.61 (0.29, 1.29)	0.31 (0.14, 0.68) 0.22 (0.09, 0.47)	<0.01 <0.01

Abbreviations: TCGA COADREAD, the Cancer Genome Atlas (colon and rectal adenocarcinoma); GSE dataset, GEO Series dataset; MSI, Microsatellite Instable; MSS, Microsatellite stable; N, number; Q1, Q2, Q3 and Q4, quartiles. Note: Only stages 2–4 were included in all the studies. The TCGA COADREAD study included both colon and rectal cancer cases. GSE17538 included colon cancer cases only. GSE39582 is not included because this dataset did not have information about disease-specific deaths. The number of cases in each study in Table 3 is lower than in the corresponding study in Table 2 because the information about disease-specific death was not available for all individuals. ^1^ Model 1: unadjusted. ^2^ Model 2: adjusted for stage at diagnosis, age and sex. ^3^ Model 3: adjusted for stage at diagnosis, age, sex, and MSI/MSS status. For the TCGA analysis of individuals with CRC, this model was also adjusted for CRC subsite (colon/rectal). MSI/MSS status was not available for GSE17538. The total number of cancer cases is lower in this model than in Models 1 and 2 in the corresponding analyses because of missing data on MSI/MSS status and CRC subsite. ^4^ *p*-trend was computed by including CFTR quartiles as an ordinal variable into the corresponding model.

**Table 4:** Somatic mutations in the *CFTR* gene in the TCGA COADREAD dataset ^1.^

CRC Cases	Cases with Mutation Data	Cases with *CFTR* Mutations			
No. of Cases	300	17			
**Somatic *CFTR* Mutations ^2^**	**Total No. of Mutations**	**Stop Loss or Gain**	**Frameshift**	**Splice Site**	**Missense**
No. of mutations	22	4	3	1	14
CF-causing mutations in CFTR2 ^3^		2			
**CRC subtypes of cases with mutations**	**^4^ Cases with subtype data**	**CIN**	**MSI**	**POLE**	
No. of cases	17	4	6	7	
Ave. no. of mutations per case		97	1665	5665	

^1^ This study was limited to cases with primary tumors, *CFTR* expression reported, diagnosed at stages 2–4, and mutation status reported. ^2^ Only loss or gain of stop codon, frame shift, splice site and missense mutations in protein coding regions were included. ^3^ The CFTR2 database (https://cftr2.org/, accessed on 21 January 2023) maintains a comprehensive list of CF-causing *CFTR* variants for the CF community. ^4^ CRC subtypes refer to clinically relevant subtypes based on mechanism of genomic instability. CIN, chromosomal instable; MSI, microsatellite instable; POLE, mutation in polymerase epsilon.

## Data Availability

All datasets analyzed in this study are publicly available. These data can be found at http://www.cbioportal.org/: data folder “Coadread_tcga_pan_can_atlas_2018; and NCBI GEO databases GSE17538 and GSE39582” (accessed on 27 September 2020, 20 June 2021 and 20 June 2021 respectively).

## Supplementary Materials

The following supporting information can be downloaded at: https://www.mdpi.com/article/10.3390/cancers15030989/s1, Table S1: *CFTR* mRNA expression in the TCGA COADREAD, GSE17538, and GSE39582 studies; Table S2: Hazard ratio (HR) and 95% confidence interval (CI) for overall and disease-specific death in relation to *CFTR* mRNA expression, presented as quartiles in individuals with colon cancer in the TCGA COADREAD study; Table S3: Hazard ratio (HR) and 95% confidence interval (CI) for overall death in relation to *CFTR* mRNA expression, dichotomized at median among individuals with CRC/colon cancer in TCGA COADREAD, GSE39582, and GSE17538 studies; Table S4: Meta-analysis: Hazard ratios (HRs) and 95% confidence interval (CI) for overall death of individuals with CRC in relation to C*FTR* expression (dichotomized at median) stratified by stage at diagnosis, age, sex, and MSI/MSS status; Table S5: Hazard ratio (HR) and 95% confidence interval (CI) for disease-specific death in relation to *CFTR* mRNA expression, dichotomized at median among individuals with CRC in the TCGA COADREAD and GSE17538 studies; Table S6: Meta-analysis: HRs (95% CI) for disease-specific death in relation to C*FTR* expression (dichotomized at median) stratified by stage at diagnosis, age and sex.
