# Supplementary material for: Lower Expression of CFTR Is Associated with Higher Mortality in a Meta-Analysis of Individuals with Colorectal Cancer
Source: Cancers (Basel). 2023 Feb 3;15(3):989. doi: 10.3390/cancers15030989 (PMC9913301; doi:10.3390/cancers15030989)
Supplement: Supplementary file 1 [file cancers-15-00989-s001.zip › cancers-2165223-supplementary.pdf]

Supplementary Table S1. *CFTR* mRNA expression[1] in the TCGA COADREAD, GSE17538, and GSE39582 studies

**Characteristics**	**TCGA COADREAD N=453**	**GSE17538 N=204**	**GSE39582 N=520**
**CFTR mRNA expression; median z-score (range)**			
Overall	-0.06 (-1.54 – 4.41)	0.29 (-3.13 – 1.48)	0.28 (-4.31 – 1.62)
By stage			
2	0.02 (-1.54 – 4.41)	0.38 (-2.93 – 1.47)	0.29 (-4.31 – 1.49)
3	-0.15 (-1.54 – 2.60)	0.17 (-2.80 – 1.48)	0.31 (-3.58 – 1.62)
4	-0.01 (-1.49 – 3.37)	0.31 (-3.13 – 1.40)	0.18 (-3.14 – 1.16)
P-value[2]	0.11	0.26	0.76

[1] *CFTR* mRNA expression was log transformed and z-score normalized in each study (mean=0, standard deviation=1).

[2] *p*-values were calculated using Mood's Median Test.

Supplementary Table S2. Hazard ratio (HR) and 95% confidence interval (CI) for overall and disease-specific death in relation to *CFTR* mRNA expression, presented as quartiles in individuals with colon cancer in the TCGA COADREAD study

**Dataset**	**HR (95% CI) across quartiles (Q1 is reference)**				***P*-trend[4]**
**TCGA COADREAD, Colon cancer cases**					
CFTR expression	Q1: -1.54 – (-0.75)	Q2: -0.72 – (-0.07)	Q3: -0.06 – 0.49	Q4: 0.49 – 4.41	
Overall death (N)	22	22	13	16	
Person-years (months)	1988	2279	2186	1903	
Model 1[1] (N = 269)	1.00 (Reference)	0.92 (0.51, 1.67)	0.53 (0.27, 1.06)	0.76 (0.40, 1.45)	0.18
Model 2[2] (N = 269)	1.00 (Reference)	1.00 (0.54, 1.85)	0.41 (0.19, 0.85)	0.90 (0.45, 1.78)	0.24
Model 3[3] (N = 269)	1.00 (Reference)	0.91 (0.46, 1.79)	0.37 (0.17, 0.81)	0.80 (0.38, 1.71)	0.20
**TCGA COADREAD, Colon cancer cases**					
CFTR expression	Q1: -1.54 –(-0.75)	Q2: -0.72 – (-0.07)	Q3: -0.06 – 0.49	Q4: 0.49 – 3.23	
Disease-specific death (N)	13	10	11	10	
Person-years (months)	1905	2167	2093	1689	
Model 1[1] (N = 255)	1.00 (Reference)	0.73 (0.32, 1.67)	0.75 (0.34, 1.67)	0.86 (0.38, 1.96)	0.70
Model 2[2] (N = 255)	1.00 (Reference)	0.60 (0.26, 1.40)	0.44 (0.19, 1.06)	0.77 (0.32, 1.84)	0.40
Model 3[3] (N = 255)	1.00 (Reference)	0.48 (0.19, 1.17)	0.35 (0.14, 0.87)	0.60 (0.24, 1.51)	0.28

Abbreviations: TCGA COADREAD, the Cancer Genome Atlas (Colon and rectal adenocarcinoma); GSE dataset, Genomic Spatial Event dataset; MSI, Microsatellite Instability; MSS, Microsatellite stability; N, number; Q1, Q2, Q3 and Q4, quartiles.
Note: Only stages 2-4 were included in these studies. TCGA COADREAD included both colon and rectal cancer cases. These analyses are of colon cancer cases only. The analysis for rectal cancer was not conducted because of small number of cases/deaths.
[1] Model 1: unadjusted.
[2] Model 2: adjusted for stage at diagnosis, age and sex.
[3] Model 3: adjusted for stage at diagnosis, age, sex, and MSI/MSS status. For the TCGA analysis of individuals with CRC, this model was also adjusted for CRC subsite (colon/ rectal). MSI/MSS status was not available for GSE17538. The total number of cancer cases is lower in this model than in models 1 and 2 in the corresponding analyses because of missing data on MSI/MSS status and CRC subsite.
[4] *p*-trend was computed by including CFTR quartiles as an ordinal variable into the corresponding model.

Supplementary Table S3: Hazard ratio (HR) and 95% confidence interval (CI) for overall death in relation to *CFTR* mRNA expression, dichotomized at median among individuals with CRC/colon cancer in TCGA COADREAD, GSE39582, and GSE17538 studies

**Study**	**HR (95% CI) for overall death**	
	Low (reference)	High
**TCGA COADREAD** (N =453)		
CFTR expression	-1.54 – (-0.07)	-0.06 – 4.41
No. of cases/deaths	226/60	227/44
Person-months	6410	6670
Model adjusted for stage at diagnosis, age and sex	1.00 (ref)	0.73 (0.49, 1.09)
**GSE17538** (N = 204)		
CFTR expression	-3.13 – 0.28	0.29 – 1.48
No. of cases/deaths	102/51	102/38
Person-months	4078	5138
Model adjusted for stage at diagnosis, age and sex	1.00 (ref)	0.57 (0.37, 0.87)
**GSE39582** (N = 520)		
CFTR expression	-3.13 – 0.28	0.29 – 1.48
No. of cases/deaths	260/97	260/84
Person-months	14,179	15,793
Model adjusted for stage at diagnosis, age, and sex	1.00 (ref)	0.76 (0.56, 1.01)

.

Supplementary Table S4: Meta-analysis: Hazard ratios (HRs) and 95% confidence interval (CI) for overall death of individuals with CRC in relation to C*FTR* expression (dichotomized at median) stratified by stage at diagnosis, age, sex, and MSI/MSS status

**Stratified variables**		**Overall death**			
		**No. of cases**	**No. of deaths**	**HR (95% CI)[1] for overall death**	***P* for interaction**
**Age, dichotomized at median**	31 – 67 y	601	153	0.70 (0.51, 0.97)	0.57
	68 – 90 y	576	221	0.79 (0.60, 1.04)	
**Sex**	Female	545	162	0.65 (0.47, 0.90)	0.65
	Male	632	212	0.72 (0.54, 0.95)	
**Stage at diagnosis**	Stage 2	537	122	0.71 (0.49, 1.02)	0.96
	Stage 3	444	132	0.67 (0.47, 0.95)	
	Stage 4	196	120	0.70 (0.48, 1.01)	
**MSI/MSS[2]**	MSS	793	237	0.69 (0.54, 0.90)	0.06
	MSI	126	31	2.72 (0.68, 10.78)	

[1]Adjusted for stage at diagnosis, age and sex. Low *CFTR* expression is a reference.

[2] The information about MSS/MSI was available for overall death in TCGA COADREAD and GSE39582, but not in GSE17538.

Supplementary Table S5: Hazard ratio (HR) and 95% confidence interval (CI) for disease-specific death in relation to *CFTR* mRNA expression, dichotomized at median among individuals with CRC in the TCGA COADREAD and GSE17538 studies

**Dataset**	**HR (95% CI) for disease-specific death**[1]	
	Low	High
**TCGA COADREAD**		
CFTR expression	-1.54 – (-0.07)	-0.06 – 3.49
No. of cases/disease-specific (CRC) deaths	214/33	219/32
Person-months	6095	6265
Analysis 1	1.00 (ref)	0.87 (0.53, 1.42)
Analysis 2 (non-CRC deaths were considered as competing events)	1.00 (ref)	0.89 (0.54, 1.45)
**GSE17538**		
CFTR expression	-2.80 – 0.27	0.29 – 1.48
No. of cases/disease-specific deaths	70/31	83/23
Person-months	2775	4194
Analysis 1	1.00 (ref)	0.49 (0.23, 0.73)
Analysis 2 (non-CRC deaths were considered as competing events)	1.00 (ref)	0.42 (0.23, 0.75)

Note: GSE39582 did not have information about causes of death, and was not included

[1]Adjusted for stage at diagnosis, age and sex.

Supplementary Table S6: Meta-analysis: HRs (95% CI) for disease-specific death in relation to C*FTR* expression (dichotomized at median) stratified by stage at diagnosis, age and sex

				**Analysis 1**[1] (non-CRC deaths were censored)		**Analysis 2**[1] (non-CRC deaths were considered as competing events)	
**Stratified variables**		**No. of cases**	**No. of deaths**	**HR (95% CI) for CRC death**	***P* for interaction**	**HR (95% CI) for CRC death**	***P* for interaction**
**Age, dichotomized at median**	31 – 67 y	300	58	0.55 (0.32, 0.95)	0.53	0.55 (0.32, 0.94)	0.46
	68 – 90 y	286	61	0.70 (0.41, 1.19)		0.73 (0.44, 1.23)	
**Sex**	Female	278	50	0.42 (0.23, 0.77)	0.09	0.41 (0.21, 0.80)	0.09
	Male	308	69	0.82 (0.50, 1.33)		0.84 (0.52, 1.36)	
**Stage at diagnosis**	Stage 2	255	20	0.63 (0.25, 1.62)	0.78	0.62 (0.24, 1.58)	0.76
	Stage 3	213	37	0.73 (0.37, 1.41)		0.74 (0.38, 1.42)	
	Stage 4	118	62	0.59 (0.35, 0.99)		0.58 (0.35, 0.97)	

[1]Adjusted for stage at diagnosis, age and sex. Low *CFTR* expression is the reference.
